# Optimized Synthesis of Small and Stable Silver Nanoparticles Using Intracellular and Extracellular Components of Fungi: An Alternative for Bacterial Inhibition

**DOI:** 10.3390/antibiotics11060800

**Published:** 2022-06-14

**Authors:** Elvira Ivonne Murillo-Rábago, Alfredo R. Vilchis-Nestor, Karla Juarez-Moreno, Luis E. Garcia-Marin, Katrin Quester, Ernestina Castro-Longoria

**Affiliations:** 1 Department of Microbiology, Center for Scientific Research and Higher Education of Ensenada (CICESE), Carr. Tijuana-Ensenada 3918, Zona Playitas, Ensenada 22860, Mexico; emurillo@cicese.edu.mx (E.I.M.-R.); luisgm@cicese.edu.mx (L.E.G.-M.); 2 Sustainable Chemistry Research Joint Center UAEM—UNAM (CCIQS) Carr. Toluca-Atlacomulco Km 14.5, San Cayetano, Toluca 50200, Mexico; arvilchisn@uaemex.mx; 3 Center for Applied Physics and Advanced Technology, UNAM, Blvd. Juriquilla 3001, Juriquilla La Mesa, Juriquilla, Queretaro 76230, Mexico; kjuarez@fata.unam.mx; 4 Center for Nanoscience and Nanotechnology, UNAM, Carr. Tijuana-Ensenada Km 107, Ensenada 22860, Mexico; quester@ens.cnyn.unam.mx

**Keywords:** silver nanoparticles, green synthesis, *Trichoderma harzianum*, *Ganoderma sessile*, bacterial inhibition, cytotoxicity

## Abstract

Silver nanoparticles (AgNPs) represent an excellent option to solve microbial resistance problems to traditionally used antibiotics. In this work, we report optimized protocols for the production of AgNPs using extracts and supernatants of *Trichoderma harzianum* and *Ganoderma sessile*. AgNPs were characterized using UV-Vis spectroscopy and transmission electron microscopy, and the hydrodynamic diameter and Z potential were also determined. The obtained AgNPs were slightly larger using the fungal extract, and in all cases, a quasi-spherical shape was obtained. The mean sizes of AgNPs were 9.6 and 19.1 nm for *T. harzianum* and 5.4 and 8.9 nm for *G. sessile* using supernatant and extract, respectively. The AgNPs were evaluated to determine their in vitro antibacterial effect against *Escherichia coli, Pseudomonas aeruginosa* and *Staphylococcus aureus*. The minimum inhibitory concentration (MIC) was determined, and in all cases the AgNPs showed an antimicrobial effect, with a MIC varying from 1.26–5.0 µg/mL, depending on the bacterial strain and type of nanoparticle used. Cytotoxicity analyses of AgNPs were carried out using macrophages and fibroblast cell lines. It was determined that the cell viability of fibroblasts exposed for 24 h to different concentrations of AgNPs was more than 50%, even at concentrations of up to 20 µg/mL of silver. However, macrophages were more susceptible to exposure at higher concentrations of AgNPs as their viability decreased at concentrations of 10 µg/mL. The results presented here demonstrate that small AgNPs are obtained using either supernatants or extracts of both fungal strains. A remarkable result is that very low concentrations of AgNPs were necessary for bacterial inhibition. Furthermore, AgNPs were stable for more than a year, preserving their antibacterial properties. Therefore, the reported optimized protocol using fungal supernatants or extracts may be used as a fast method for synthesizing small AgNPs with high potential to use in the clinic.

## 1. Introduction

The production of nanoparticles (NPs), with a scale of 0.1 to 100 nm in at least one of its three dimensions [1], has allowed the manufacture of a variety of nanomaterials for their use in optical, magnetic, electrical, as well as in the food, cosmetic, pharmaceutical and diagnostic industries, among others [2,3]. Silver NPs (AgNPs) in particular have important biomedical applications; they have been evaluated as antimicrobial agents in dentistry and surgical materials, as well as in cancer diagnosis and therapy [2,4,5,6,7]. AgNPs are regularly produced by chemical or physical methods, which are associated with high environmental impact due to the use of toxic chemicals, high-energy consumption and the use of high temperatures during their manufacture [3]. The most common chemical method is the chemical reduction, using three main components: metal precursors, reducing agents and stabilizing/capping agents. The advantage of chemical methods is the high yield, compared to physical methods; however, the use of toxic and hazardous materials is a clear disadvantage. The most important physical methods are evaporation-condensation and laser ablation. Here, the clear advantage is that no hazardous chemicals are involved, but these methods have low yield and a high energy consumption [3].

In the past decades, the synthesis of NPs using biological materials has been extensively studied because it has been recognized as a simple and rapid method, which allows obtaining non-toxic, eco-friendly organic molecules to produce metallic NPs under optimized conditions [3,8,9]. Among the biological materials that have been reported for the synthesis of NPs, fungi are considered excellent candidates because they secrete a large number of extracellular enzymes and secondary metabolites, which serve as bio-reducing and stabilizing agents for the production of metallic NPs.

Biogenic AgNPs are potentially useful in the clinic; they have been proposed as antimicrobial agents to offer an alternative treatment to solve problems of resistance to antibiotics traditionally used against pathogenic strains [10,11]. However, for the application of biogenic NPs in the medical area, it is necessary that the biological material used for the synthesis does not come from pathogenic microorganisms, containing potentially toxic molecules for humans [10].

The biosynthesis of NPs using living microorganisms such as fungi can occur intracellular or extracellularly [11,12]. Although the exact mechanism for the synthesis of nanoparticles has not been fully described, some well-described mechanisms have been proposed to intervene in the process. Some of the suggested mechanisms are an electron transfer dependent on enzymatic processes, electron charges, reducing agents and oxidative stress, among others, where the metal ions are converted into a less toxic form in the fungal mycelium [11,13]. On the other hand, the use of extracts and/or supernatants excreted by the mycelium has also been described for the biogenic synthesis of NPs [8]. Here, the biosynthesis process is dependent on the presence of bioactive metabolites, which intervene in the synthesis and stabilization of NPs [14,15,16]. According to the reports, each of these compounds can influence the particular characteristics of the obtained NPs, their properties and their potential applications [15,16].

Most of the studies for the biogenic synthesis of metallic NPs using fungi report the use of extracellular components [8]. The extracellular or supernatant fraction of fungal liquid cultures contain extracellular proteins and other secreted metabolites, while the intracellular components contain the released proteins, enzymes, primary and secondary metabolites, DNA and RNA fragments, etc., after mechanically disrupting the fungal washed biomass [17]. The use of both intracellular and extracellular components has only been reported for several thermophilic fungi [17] and various basidiomycetes [18]. Differences in size and polydispersity were observed when using the intracellular and extracellular fractions of fungal liquid cultures. Bigger and polydisperse NPs are produced when using the intracellular components, compared with smaller polydisperse NPs when using the extracellular fraction [17]. In addition, shape, size and aggregation of synthesized nanoparticles depend on the species used and the type of reduction agent, i.e., extracellular and intracellular fractions [17,18].

However, one of the main challenges when using biogenic precursors is to achieve the reproducibility of the synthesis method [19], which is essential to be able to reproduce the specific characteristics and properties of the synthesized nanoparticles.

Therefore, the objective of this study is to obtain and define a reproducible protocol for the biosynthesis of small silver NPs (1–20 nm), using extracts and supernatants of fungi and compare their antibacterial and biocompatibility properties. For this, *T. harzianum* and *G. sessile* were used since they are considered not harmful for humans. *Trichoderma* spp. are commonly used to stimulate plant growth and for the bio-control of phytopathogenic fungi in economically important crops [20]. *Ganoderma* spp. are wood degrading fungi, and their crude extracts are traditionally used and consumed worldwide because of their antioxidant and immune-stimulant properties [21]. Therefore, toxic or harmful effects due to the biological material used for the synthesis were prevented. After defining a reproducible protocol, the synthesized AgNPs were evaluated in in vitro assays to determine their antimicrobial effect against pathogenic bacteria. In addition, their biocompatibility in murine cell models of fibroblasts and macrophages was determined.

## 2. Materials and Methods

### 2.1. Strains and Culture Conditions

Strains of *Ganoderma sessile* and *Trichoderma harzianum* were obtained from the stock of the Microbiology Department of the Ensenada Center for Scientific Research and Higher Education (CICESE). Strains were cultured in potato dextrose agar (PDA) in Petri plates at 26 °C and stocks conserved at 4 °C.

To obtain fungal biomass in liquid cultures, the strains were cultivated in potato-dextrose broth (PDB) and incubated at 26 °C under gentle agitation in an orbital shaker (Orbit Environ Shaker) at 125 rpm for 7 days. To inoculate the liquid cultures, plugs from fresh Petri plate cultures were placed in 500 mL flasks containing 300 mL of medium. After incubation, biomass was collected and weighted.

### 2.2. Fungal Extracts

To obtain the intracellular components of fungal strains, the biomass of each fungus was separated from the culture broth, washed with distilled water and triturated in an agate mortar with deionized water in a ratio of 1:1 (g/mL) until obtaining a homogeneous mixture [22]. Afterward, the resulting mixture was centrifuged at 10,000 rpm for 15 min, the pellet was discarded, and the aqueous extract was filtered with a 0.22 µm nitrocellulose membrane to eliminate any biomass residues.

### 2.3. Fungal Supernatants

To obtain the extracellular components of fungal strains, the biomass of each fungus was washed with deionized water, weighed and placed in a flask with 100 mL of deionized water. Cultures were maintained at 26 °C for 3 days in a shaker at 125 rpm. Afterward, biomass was removed by filtration and the resulting supernatant was centrifuged and subsequently filtered with a 0.22 µm nitrocellulose membrane.

### 2.4. Biosynthesis of Silver Nanoparticles (AgNPs)

The optimized protocol for the synthesis of AgNPs was carried out with the supernatants and extracts of *T. harzianum* and *G. sessile*. Final conditions, according to the results previously obtained (Supplementary Materials), were as follows: Supernatant was obtained using 10 g of previously washed biomass that was incubated in 100 mL of deionized water for 3 days. Fungal extracts were obtained using the protocol reported by Quester et al. [22]. The formation of NPs was best achieved using a ratio of 1:3 *v*/*v* of (extract or supernatant)/1 mM AgNO_3_ and incubation for 3 days at 60 °C.

### 2.5. UV-Vis Spectroscopy

Each synthesis was evaluated by UV-Vis spectroscopy at 300 to 700 nm after 24 h; at this time the color of the reaction changed from clear to pale brown, which is characteristic of the reaction kinetics in the synthesis of AgNPs. Curves of absorbance for the optimized protocol are reported after 72 h of incubation; at this time, no further change in absorbance was detected.

### 2.6. Transmission Electron Microscopy

To determine the size and shape of synthesized nanoparticles, 5 µL of the samples were placed on formvar/carbon-coated copper grids and allowed to dry. Samples were analyzed under transmission electron microscopy (TEM) (Hitachi H7500, Hitachi Ltd. Tokyo, Japan) at 100 kV. Samples from the optimized protocol were placed on Lacey carbon copper grids and analyzed under high-resolution transmission electron microscopy (HRTEM) (JEM0-2100 from JEOL, JEOL Ltd. Tokyo, Japan) at 200 kV to obtain the crystalline structure of the particles.

### 2.7. Dynamic Light Scattering (DLS) Characterization

For optimized synthesis, the zeta potential and the hydrodynamic diameter of the synthesized AgNPs were measured with a Zetasizer Nano ZS instrument (Malvern Panalytical Inc., Westborough, MA, USA).

### 2.8. FTIR Analysis of Synthesized AgNPs

To determine the functional groups from the supernatants/extracts involved in the reduction and stabilization of silver nanoparticles, the samples were placed on a glass slide and allowed to dry at room conditions and finally the Fourier transform infrared spectroscopy (FTIR) spectra were collected using a Bruker Tensor 27 FT-IR spectrometer (SpectraLab Scientific Inc., Markham, ON, Canada) with resolution of 0.5 cm^−1^ in transmission mode. For each spectrum, an average of 5 scans was recorded over the 450–4000 cm^−1^ wavelength range.

### 2.9. Evaluation of Antibacterial Effect

Minimum inhibitory concentration (MIC) and minimum bactericidal concentration (MBC) was evaluated with AgNPs obtained with the optimized protocol. Amount of silver was calculated for serial dilutions and approximate concentrations were used (0.31, 0.63, 1.2, 2.5, 5.1, 10.1, 20.2 and 40.4 µg/mL). The bacteria strains used in this study were *Staphylococcus aureus* (ATCC 25923), *Pseudomonas aeruginosa* (ATCC 27853) and *Escherichia coli* (ATCC 25922). To prepare the inoculum, fresh Petri plate cultures were used. Each microorganism was suspended separately in sterile saline solution (0.9% NaCl) and adjusted by UV-Vis spectroscopy to an optical density (OD) corresponding to a 0.5 McFarland standard (1.5 × 10^8^ CFU / mL).

For the inhibition assays, the Mueller Hinton liquid culture medium was used. The bacteria were cultured in 96-well plates at 37 °C for 24 h. To evaluate qualitatively and quantitatively the degree of inhibition in bacterial growth, bacteria were exposed to serial dilutions of the produced AgNPs according to concentrations mentioned above. In each well, 50 µL of the culture medium of the corresponding strain was placed and 50 µL of the corresponding AgNP dilution was added. Samples were incubated for 24 h, and then 2.5 µL of the sample was taken from each culture and spread on Mueller Hinton agar medium and incubated at 37 °C for 24 h. As negative controls, the supernatants and extracts of each AgNP synthesis were used. These studies were performed in triplicate at different times for each strain. For qualitative evaluation, changes in coloration and turbidity in the culture media were taken into account, as well as the presence of colony growth when the cultures were spread on agar plates.

#### 2.9.1. Cell Viability Assay

Bacteria were seeded in a 96-well plate for 24 h at 37°C in the presence of different silver concentrations (0.31, 1.26, and 10 μg/mL) or with the supernatant from *T. harzianum* (TS) or *G. sessile* (GS), in a final volume of 200 μL per well. After this, cells were washed thrice with PBS 1x and then 10 μL of MTT (3-(4,5-dimethyl-2-thiazolyl)-2,5-diphenyl-2H-tetrazolium bromide, 0.5 μg/μL) was added to each well in 90 μL LB media and incubated in darkness for 4 h at 37°C. The reduction of MTT was used to assess cell viability, as reported by [23]. Briefly, the absorbance of the samples was read in an ELISA plate reader (Thermo Fisher Scientific, Waltham, MA, USA). The background absorbance of the cell viability test was measured at 690 nm and subtracted from the absorbance values at 570 nm. A control from cell viability was taken from cells cultivated in LB media without treatment, while positive control was carried out by exposing the cells to Ciprofloxacin at a concentration of 50 μg/mL. The absorbance of negative control cells was used to calculate cell viability from the obtained data from three experiments.

#### 2.9.2. Reactive Oxygen Species (ROS) Quantification by Fluorimetry

Cells were seeded in a 96-well plate at a concentration of 1 × 10^5^ cells per well and incubated for 24 h at 37 °C, with different silver concentrations (0.31, 1.26, and 10 μg/mL) and with the supernatant from *T. harzianum* (TS) or *G. sessile* (GS). After this, cells were washed thrice with 200 μL of PBS 1× and incubated in darkness with DCFDA (2′,7′-dichlorofluorescein diacetate, 45 μM) for 60 min at 37°C. Then the fluorescence was recorded with a Cary Eclipse fluorescence spectrophotometer (Agilent Technologies CA, USA) using a 485 nm excitation laser and 530 nm emission laser. Bacteria without silver concentrations were used as a negative control, while cells incubated with 1mM of H_2_O_2_ were considered positive for generating higher ROS levels. The results were plotted by comparing the mean ± standard deviation fluorescence of three experiments with the positive control of ROS generation (H_2_O_2_).

### 2.10. Biocompatibility Evaluation

To evaluate the biocompatibility of AgNPs obtained with optimized protocol, cell viability tests were assessed in the cell lines from L929 fibroblasts and RAW 264.7 macrophages. Cytotoxicity assays were performed using the standard MTT metabolic reduction method, based on the reduction of MTT. The cell lines were obtained from ATCC and cultured in Dulbecco’s Modified Eagle’s Medium (DMEM) supplemented with 10% fetal bovine serum, 1% L-glutamine, 1% antibiotic/antifungal and 1.5 g of sodium bicarbonate. Cells grown in DMEM medium without AgNPs were used as a positive control, and as negative viability control, cells were incubated with 1% TritonX-100 in PBS to induce cell death (negative viability control). For cytotoxicity assays, dilution series of biogenic AgNPs were carried out, according to the results obtained in the bacterial inhibition assays. The cytotoxicity of the precursors was also measured (AgNO_3_ and reducing agents). For the assays, 10,000 cells were seeded per well in a 96-well plate and incubated for 24 h at 37 °C and at atmosphere of 5% CO_2_. Subsequently, the different concentrations of AgNPs were added in a final volume of 100 µL per well and incubated for 24 h at 37 °C and in an atmosphere of 5% CO_2_. Afterward, the medium was removed and the cells were washed three times with 200 µL of PBS. Thereafter, 10 µL of MTT (0.25 mg/mL) and 90 µL of DMEM medium were added; the plates were incubated for 4 h at 37 °C and an atmosphere of 5% CO_2_. Finally, 100 µL of isopropanol were added to dissolve the formazan, a product of MTT metabolism. The absorbance of the culture plates was read at 570 and 690 nm. The resulting values were taken as an indirect measure of cell viability, taking the value of absorbance of cells grown in DMEM media as 100% of live cells. Three independent assays were conducted.

### 2.11. Statistical Analysis

Mean and standard deviation of nanoparticle size were calculated after measuring 1000 nanoparticles of each synthesis. Inhibition and biocompatibility experiments were carried out in triplicate and were expressed as the mean and standard deviation.

## 3. Results

### 3.1. Optimized Synthesis of AgNPs Using Fungal Extract and Supernatant of T. harzianum and G. sessile

**Figure 1:**
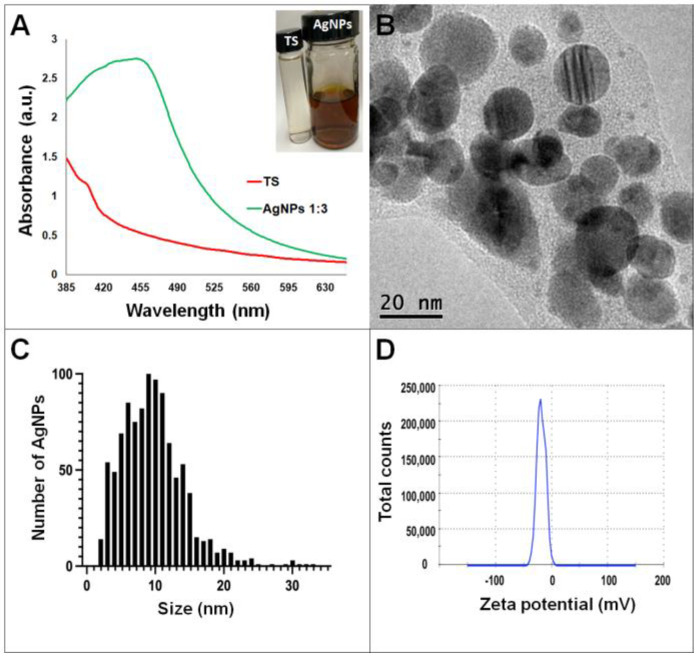
Silver nanoparticles obtained with the optimized protocol using the supernatant of *T. harzianum* (**A**) UV-Vis analysis of AgNPs-TS after 72 h of reaction, (**B**) TEM micrograph showing the morphology of NPs, (**C**) size distribution histogram of AgNPs, (**D**) Zeta potential. Inset in (**A**) shows the fungal supernatant and synthesized NPs. TS = *Trichoderma* supernatant.

**Figure 2:**
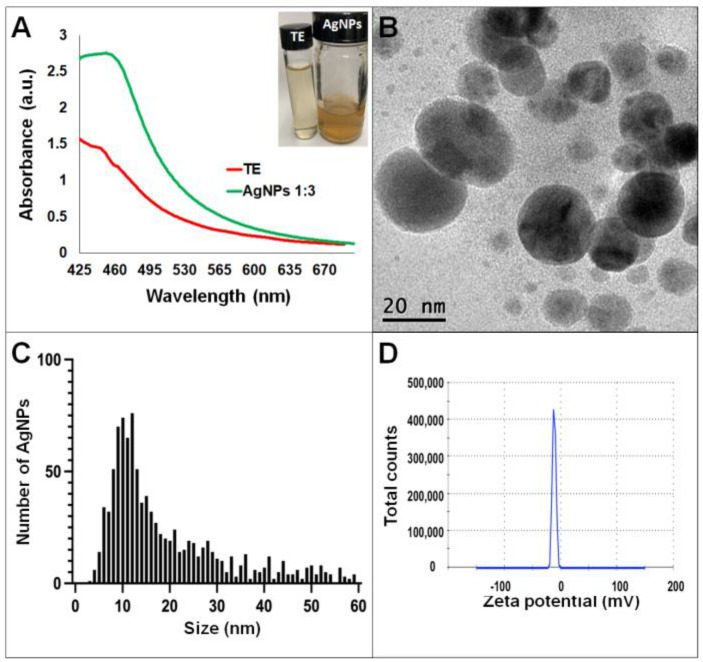
Silver nanoparticles obtained with the optimized protocol using the extract of *T. harzianum* (**A**) UV-Vis analysis of AgNPs-TE after 72 h of reaction, (**B**) TEM micrograph showing the morphology of NPs, (**C**) size distribution histogram of AgNPs, (**D**) Zeta potential. Inset in (**A**) shows the fungal extract and synthesized NPs. TE = *Trichoderma* extract.

**Figure 3:**
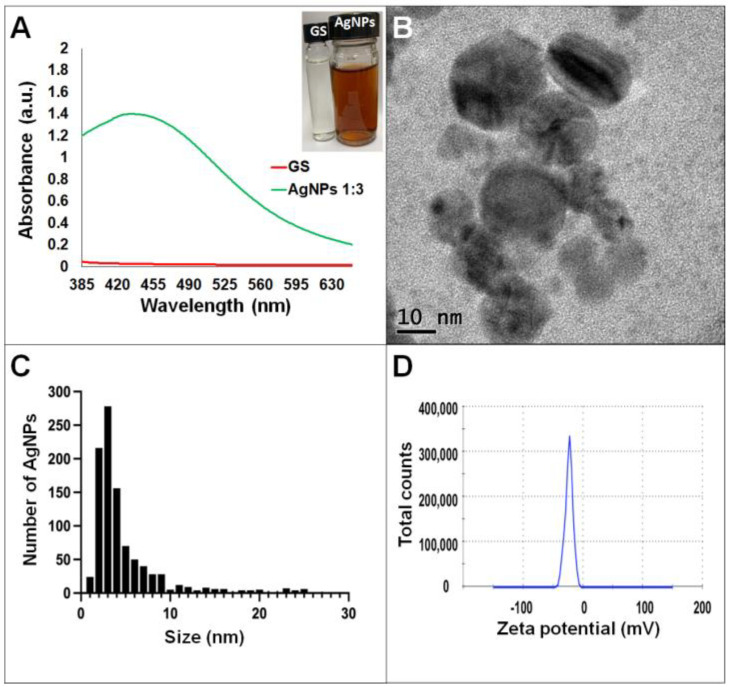
Silver nanoparticles obtained with the optimized protocol using the supernatant of *G. sessile*. (**A**) UV-Vis analysis of AgNPs-GS after 72 h of reaction, (**B**) TEM micrograph showing morphology of NPs, (**C**) size distribution histogram of AgNPs, (**D**) Zeta potential. Inset in (**A**) shows the fungal supernatant and synthesized NPs. GS = *Ganoderma* supernatant.

**Figure 4:**
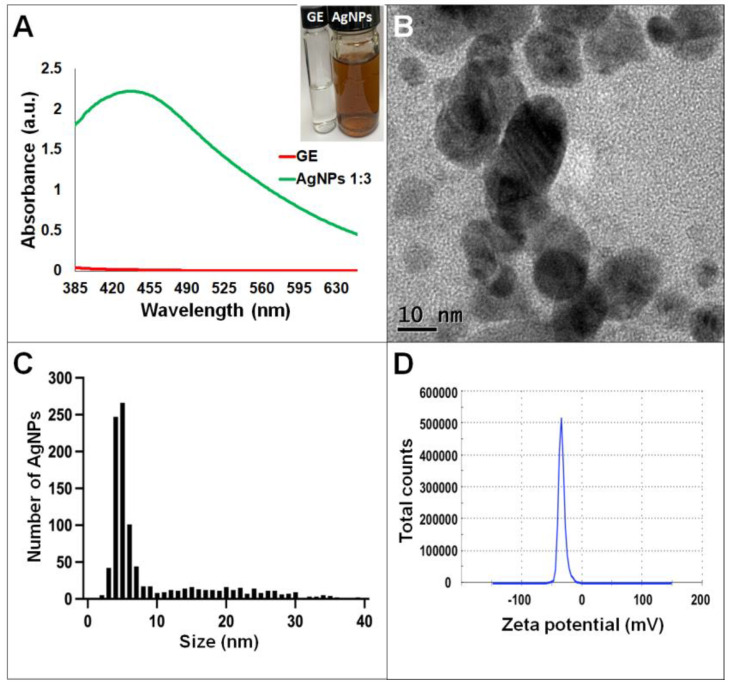
Silver nanoparticles obtained with the optimized protocol using the extract of *G. sessile*. (**A**) UV-Vis analysis of AgNPs-GE after 72 h of reaction, (**B**) TEM micrograph showing morphology of NPs, (**C**) size distribution histogram of AgNPs, (**D**) Zeta potential. Inset in (**A**) shows the fungal extract and synthesized NPs. GE = *Ganoderma* extract.

Synthesis of AgNPs in all reactions was observed after 24 h of incubation; however, it was after three days that no further change in the reaction was detected, i.e., no further change in color or agglomeration was observed and the peaks of absorbance by UV-Vis analysis were similar (values were 438 and 442 nm for *G. sessile* and 453 and 460 nm for *T. harzianum,* for AgNPs synthesized with the supernatant and extract, respectively). In all cases, the reaction changed gradually from clear/pale yellow to dark brown, and the characteristic curve of absorbance for AgNPs was obtained. Analysis by UV-Vis spectroscopy indicated the SPR (surface plasmon resonance) absorption maximum in the range of 400–500 nm (Figure 1, Figure 2, Figure 3 and Figure 4). Analysis by TEM revealed polydispersed quasi-spherical nanoparticles in all synthesis reactions (Figure 1, Figure 2, Figure 3 and Figure 4). AgNPs obtained with the supernatant of *T. harzianum* (AgNPs-TS) presented an average size of 9.6 ± 4.6 nm and size range of 1 to 33 nm (Figure 1C), with a hydrodynamic diameter of 22 nm and a zeta potential of –18.5 mV (Figure 1D). AgNPs obtained with the extract of *T. harzianum* (AgNPs-TE) were slightly larger, presenting an average size of 19.1± 12.6 nm and a size range of 3 to 59 nm (Figure 2C) with a hydrodynamic diameter of 33 nm and a zeta potential of –11.8 mV (Figure 2D). Average size for AgNPs obtained with the supernatant of *G. sessile* (AgNPs-GS) was 5.4 ± 3.0 nm with size range of 1–50 nm (Figure 3C); with a hydrodynamic diameter of 24 nm and zeta potential of –23.3 mV (Figure 3D). Nanoparticles synthesized using the extract of *G. sessile* (AgNPs-GE) presented an average size of 8.9 ± 7.7 nm and size range of 1–38 nm (Figure 4C), with a hydrodynamic diameter of 47 nm and a zeta potential of –33.2 mV (Figure 4D). Characterization of AgNPs obtained with the optimized protocol, using the supernatant and extract of both fungi is summarized in (Table 1).

**Figure 5:**
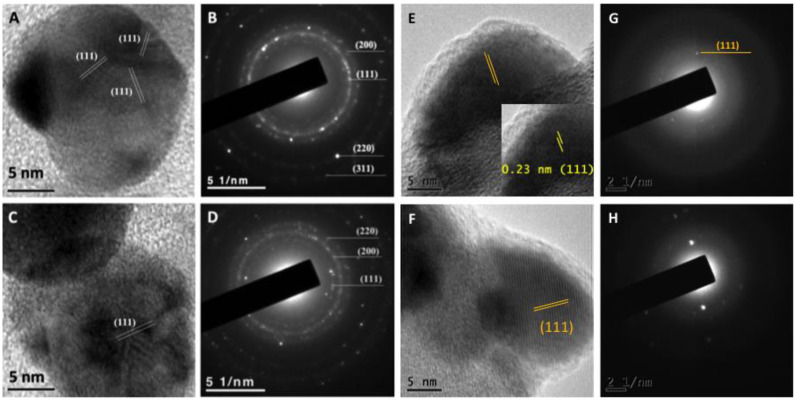
High-resolution transmission electron microscopy of AgNPs synthesized using the supernatant of *T. harzianum* (**A**) and *G. sessile* (**C**) with planes (111) and the corresponding diffraction pattern (**B**,**D**) indicating the crystalline structure. HRTEM image (**E**) and SAED pattern (**G**) of the Ag nanoparticles obtained with *T. harzianum* supernatant and HRTEM micrograph (**F**) and SAED pattern (**H**) of Ag synthetized with *G. sessile* supernatant, respectively.

After one year of synthesis, NPs synthesized using the optimized protocol were analyzed by UV-Vis and TEM, and in none of the cases aggregates or precipitation of the nanoparticles were observed. Furthermore, NPs were analyzed under high-resolution transmission electron microscopy confirming quasi-spherical shape. In addition, the lattice fringe spacing of AgNPs was measured confirming the planes (111) (Figure 5A,C). Additionally, the corresponding diffraction patterns of pure Ag particles with a crystalline structure were obtained (Figure 5B,D). HRTEM images (Figure 5E) and (Figure 5F) show the lattice spacing was about 0.23 nm between the (111) planes, consistent with FCC structure of Ag. Furthermore, the SAED patterns in Figure 5G,H indicated that the samples have a polycrystalline nature, with a d-spacing of 0.23 nm, which could be indexed as (111) reflection corresponding to FCC silver structure.

### 3.2. FTIR Analysis of Synthesized AgNPs

**Figure 6:**
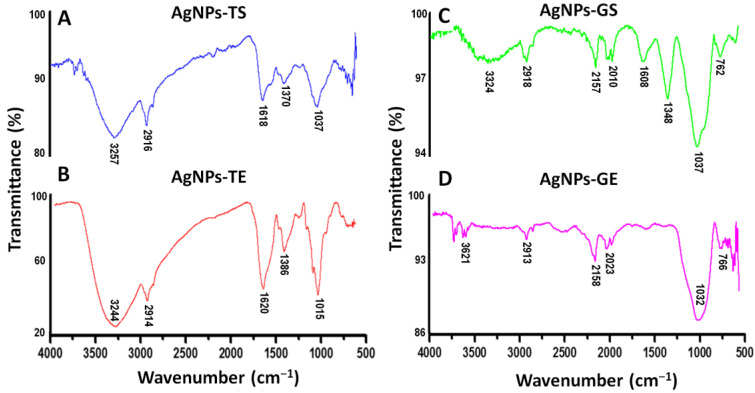
FTIR spectra of synthesized AgNPs. (**A**,**B**) AgNPs synthesized using the supernatant (TS) and extract (TE) of *T. harzianum*, respectively, (**C**,**D**) AgNPs synthesized using the supernatant (GS) and extract (GE) of *G. sessile*, respectively.

Fourier transform infrared spectroscopy (FTIR) was used to elucidate the possible functional groups of the supernatants and extracts of *T. harzianum* and *G. sessile* that are involved in the reduction and stabilization of AgNPs. Samples of the produced AgNPs were analyzed with a FTIR spectrometer in the region of 4000–500 cm^−1^. The spectrum of AgNPs using both the supernatant and extract of *T. harzianum* revealed a similar profile (Figure 6A,B). The bands around 3257 and 3244 cm^−1^ correspond to the N–H stretching vibrations, the bands at 2916 and 2914 cm^−1^ correspond to the C–H stretching vibrations. The absorption peaks at 1618 and 1620 cm^−1^ show the C=C stretching and N-H bending; the peaks at 1370 and 1386 cm^−1^ correspond to the –C–N stretching vibrations; and finally, the peaks at 1037 and 1015 cm^−1^ correspond to the C–O bending and C-N stretching.

The spectra of AgNPs using the supernatant and extract of *G. sessile* were similar; however, in AgNPs-GE the peaks at 1608 and 1348 cm^−1^ were hardly noticeable (Figure 6D). The absorption peaks at 3324 and 3621 cm^−1^, correspond to the O–H stretching vibrations. The absorption peaks at 2918 and 2913 cm^−1^ correspond to stretching vibrations of C–H bonds. The absorption peaks at 2157 and 2158 cm^−1^ correspond to C=C conjugated and C≡ stretch for alkynes. Bands at 2010 and 2023 cm^−1^ correspond to C≡C–H stretch, 1608 cm^−1^ indicates the presence C=C stretching and N-H bending vibrations. The peak at 1348 cm^−1^ indicates the presence of –C–N stretching, the strong absorption peaks at 1037 and 1032 cm^−1^ correspond to the C–O bending and C–N stretching, and the absorption peaks at 762 and 766 cm^−1^ correspond to the C–H (rocking) and N–H rocking vibrations.

The spectrum of the AgNPs synthesized with the extracts showed more intense peaks; this could be due to the presence of a higher concentration of biomolecules. In general, FTIR spectra of AgNPs synthesized using supernatant and extract of the same fungal species were similar, while some band assignments were not detected in both species (Table 2).

### 3.3. Evaluation of the Bactericidal Properties of AgNPs

**Figure 7:**
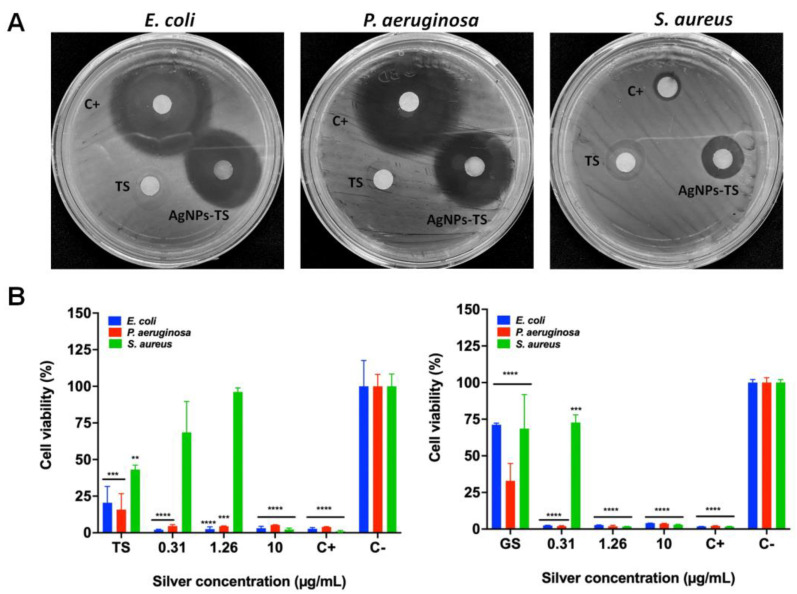
Representative images of bacterial inhibition strains with biosynthesized AgNPs (**A**) and cell viability of bacteria (**B**) after exposed to AgNPs for 24 h. Results are expressed as the mean ± SD (n = 3) ** *p* < 0.01; *** *p* < 0.001; **** *p* > 0.0001 using two-way ANOVA with a Dunnett’s multiple comparisons tests.

**Figure 8:**
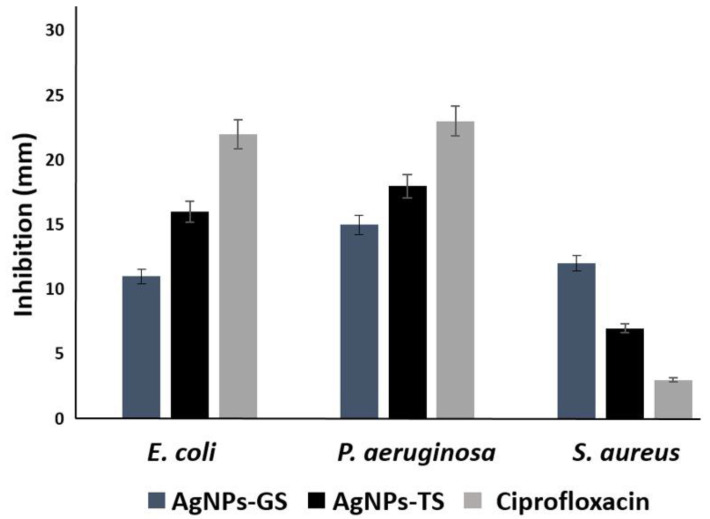
Bar graph showing zone of inhibition produced by biosynthesized AgNPs using supernatants of *G. sessile* (GS) and *T. harzianum* (TS) against *E. coli*, *P. aeruginosa* and *S. aureus*.

All bacterial strains were inhibited at low silver concentrations using the obtained AgNPs (Table 3). Inhibition of *S. aureus* was observed from concentrations of approximately 2.5 µg/mL with AgNPs-GS, the MBC was obtained at a concentration of 10.0 µg/mL with AgNPs of both fungi. In the case of *P. aeruginosa*, inhibition was obtained with AgNPs at concentrations from 1.26 µg/mL; the MBC was 2.5 µg/mL for all AgNPs. For *E. coli*, inhibition was observed also from a concentration of 1.26 µg/mL and the MBC was 2.5–5.0 µg/mL depending on the type of particle. After 12 months of storage, inhibition of all bacteria was observed using the disk diffusion method (Figure 7A), and the CMI was determined with these NPs (Table 3). Fresh supernatants were used in the disk diffusion method, but no evident inhibition was observed (Figure 7A). Inhibition with AgNPs-TS was better against *E. coli* and *P. aeruginosa*, although inhibition by Ciprofloxacin was superior to those of AgNPs, However for *S. aureus* better inhibition was obtained with AgNPs-GS, even compared with Ciprofloxacin (Figure 8).

The MTT assay also corroborated the bactericidal properties of AgNPs (Figure 7B). The growth of *E. coli* and *P. aeruginosa* was inhibited with all the silver concentrations used. However, the inhibition of *S. aureus* was only decreased in the presence of 10.0 μg/mL of silver from AgNPs obtained from the extract of *T. harzianum*. In addition, the supernatant from *T. harzianum* (TS) significantly diminished the growth of the three bacteria. In contrast, all the silver concentrations from AgNPs obtained by the extract from *G. sessile* exhibited a more prominent toxic effect for *E. coli* and *P. aeruginosa*. Indeed, the growth of *S. aureus* decreased after 1.26 μg/mL of silver. However, the supernatant from *G. sessile* (GS) induced an apparent cytotoxic effect for *P. aeruginosa* rather than for *E. coli* and *S. aureus*.

**Figure 9:**
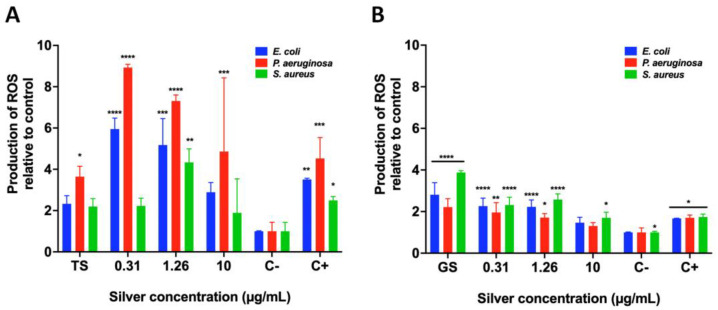
ROS production by biosynthesized AgNPs using supernatants of *T. harzianum* (**A**) and *G. sessile* (**B**) against *E. coli*, *P. aeruginosa* and *S. aureus*. TS = *T. harzianum* supernatant, GS = *G. sessile* supernatant. Results are expressed as the mean ± SD (n = 3) * *p* < 0.05; ** *p* < 0.01; *** *p* < 0.001, **** *p* < 0.0001 using two-way ANOVA with a Dunnett’s multiple comparisons tests.

The ROS production was scored to investigate whether the bactericidal effect elicited by AgNPs was due to oxidative stress. As observed in Figure 9, it is clear that overproduction of ROS correlated with the bactericidal effect. In the case of AgNPs produced with the extract from T. harzianum, all the concentrations of silver induced an abrupt overproduction of ROS in both E. coli and P. aeruginosa. However, the fact that ROS overproduction in S. aureus is less could be attributed to a growth inhibitory effect after 0.31 μg/mL of silver. In addition, the supernatant from T. harzianum (TS) did not induce any overproduction of ROS. Interestingly, all the silver concentrations used herein, from the AgNPs produced by the extract of G. sessile, also induced ROS overproduction in all the tested bacteria, especially for S. aureus; the diminishment of growth was more significant than for E. coli and P. aeruginosa. Worth mentioning is that the supernatant from G. sessile (GS) also induced overproduction of ROS in all the tested bacteria.

### 3.4. Biocompatibility Evaluation of AgNPs in Mammalian Cell Lines

For biocompatibility analysis, cell lines were exposed to AgNPs obtained from the optimized protocol using the extract and supernatant of both fungi. The concentration range evaluated was determined according to the concentration at which bacteria were inhibited, including a higher concentration of silver. Serial dilutions of AgNPs were used in all cases at the following approximate concentrations of silver: 0.0, 0.63, 1.26, 2.53, 5.06, 10.11 and 20.23 µg/mL.

**Figure 10:**
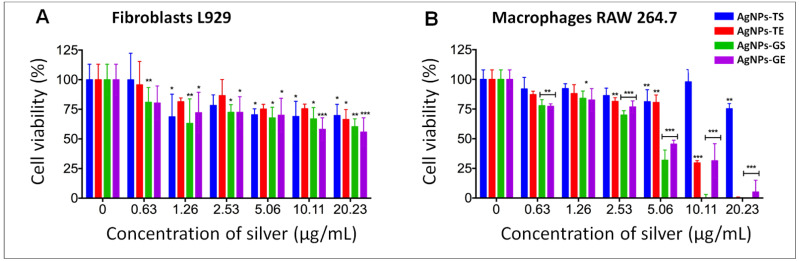
Cell viability assay in fibroblasts (**A**) and macrophages (**B**) exposed to AgNPs synthesized using the supernatant and extract of *T. harzianum* and *G. sessile*. The bars represent the mean and standard deviations of experiments performed in triplicate. * *p* ≤ 0.05, ** *p* ≤ 0.01, *** *p* ≤ 0.001.

In the cytotoxicity assays on fibroblasts, it was observed that cell viability was higher at the lowest concentration (0.63 µg/mL) of silver in cells exposed to AgNPs synthetized using both fungi. Results obtained at all other concentrations and types of NPs showed a degree of cytotoxic effect; however, cell viability was greater than 55% (Figure 10A).

In the biocompatibility tests carried out on macrophages, it was observed that in general, cell viability decreased in a dependent manner with the increase in the concentration of silver. Although macrophages were more sensitive at higher concentrations of silver, cell viability was greater than 80% in concentrations of up to 20.23 µg/mL with AgNPs synthesized with the supernatant of *T. harzianum.* Good viability also was observed at concentrations up to 5.06 µg/mL for AgNPs-TS and AgNPs-TE. However, with AgNPs-GS and AgNPs-GE cell viability decreased drastically at concentrations of 5 µg/mL and higher (Figure 10B).

## 4. Discussion

It is well-documented that silver nanoparticles (AgNPs) can be produced using extracts of numerous biological materials, searching for eco-friendly protocols. Furthermore, by using extracts of biological material to synthesize AgNPs, the potential use in medical treatments is increased since the risk of adverse reactions to harmful chemicals is avoided. Silver nanoparticles possess excellent antimicrobial properties; hence, their use in difficult-to-treat cutaneous infections could be a feasible option if the reducing agent used for the synthesis does not provoke unfavorable effects. Among the biological materials used to synthesize nanoparticles, fungi are reported as excellent reducing agents for the production of metallic nanoparticles [8]. However, few studies have reported the stability of the produced nanoparticles and the biocompatibility with animal cells. Therefore, in this work we report an optimized and highly reproducible protocol for the production of small stable AgNPs using *Trichoderma harzianum* and *Ganoderma sessile*.

The use of *T. harzianum* for the synthesis of AgNPs has been reported previously [24,25,26,27,28,29,30,31]; however, in all cases, only the extracellular supernatant has been used as a reducing agent. Using extracellular supernatants of fungi is widely applied in the synthesis of metallic nanoparticles due to the straightforward protocol; thus, it is the most common fungal reducing agent [8]. To our best knowledge, there are only two reports using the extracellular and intracellular fractions for the synthesis of metallic NPs. For instance, Molnár et al. [17] used the extracellular and intracellular components and the autolyzates of thermophilic filamentous fungi to synthesize gold nanoparticles. Although the protocols used by [17] have significant differences compared with the ones used in this work and the synthesized NPs were gold, not silver, in general terms the results are comparable; they found smaller, less polydispersed NPs using the supernatants, compared with the intracellular fractions. In this work, AgNPs synthesized using the intracellular extracts of *T. harzianum* were bigger than those obtained with the supernatants (average size of 19 and 9 nm, respectively). In addition, in most reports the supernatants used for the synthesis of nanoparticles contain traces of the culture media [17]. They demonstrated that those remnants contribute to the formation of NPs. In this work, the biomass was thoroughly washed before incubation in deionized water to obtain the supernatant, and the same was done before obtaining the extracts; therefore, only fungal compounds were responsible for the formation and stabilization of NPs. In the study of [18], the use of intracellular and extracellular components of *Lentinus edodes*, *Ganoderma lucidum*, *Pleurotus ostreatus* and *Grifola frondosa* was reported for the synthesis of silver and gold NPs. They found large and aggregated AgNPs using the extracellular fraction of all basidiomycetes; however, the culture conditions and synthesis protocol differ largely from the one used in this work.

Synthesis of metallic NPs using *G. sessile* has not been previously reported; therefore, the optimized protocol used for *T. harzianum* was applied. A similar protocol was used to synthesize NPs using *G. lucidum* by [32], who reported AgNPs of 10.72 nm using the supernatant of fruiting bodies. The authors described as optimum conditions the use of 1 mM AgNO_3_ and a reaction temperature of 85 °C. However, in the case of *G. sessile* AgNPs were approximately 50% smaller, compared to those obtained with *T. harzianum*. Differences in size can be due to the nature and/or number of excreted metabolites. In fact, differences in the production of AgNPs were detected even when using fungi of the same genera, as reported by Devi et al. [33], who explored the synthesis of AgNPs using the same protocol for different species of *Trichoderma*. Although the size range for each species was not reported, they found differences in the synthesis reaction; the highest UV-Vis absorption band was found for *T. virens* followed by *T. longibranchiatum*, *T. asperellum*, *T. pseudokoningii* and *T. harzianum*. The authors explored the role of reductases in the biosynthesis of AgNPs by the nitrate reductase assay. However, they did not find a direct relationship with the nitrate reductase activity and the production of AgNPs between species.

The analysis by DLS revealed a bigger size of nanoparticles in all cases, compared with the size obtained by TEM. This was expected since DLS measures the hydrodynamic diameter, which includes the diameter of the nanoparticle plus the layer of molecule(s) attached or absorbed on their surface, whereas the analysis by TEM provides the projected surface area of dry particles [34]. Thus, the size of particles obtained by DLS is substantially increased by the capping agents, which are bound to biological synthesized NPs [30,35]. Zeta potential (ZP) measurements were negative for all obtained AgNPs; values were similar to previously reported [30,36,37]. Considering the commonly used guidelines [34], NPs are relatively stable and moderately stable for *T. harzianum* and *G. sessile*, respectively. Values of ±30 mV are considered as highly stable; however, there are stable colloids with low ZP and vice versa, since stability depends not only on electrostatic repulsive forces but also on the van der Waals attractive forces [34]. ZP values for *G. sessile* were higher compared with those of *T. harzianum;* this could be due to the nature of the reducing agent, since ZP is influenced by several parameters such as pH, ionic strength and concentration [34]. Nevertheless, NPs did not display agglomeration even after one year of storage at room temperature.

FTIR spectroscopy provides information about the organic functional groups attached to AgNPs, which can be responsible for their synthesis and/or stabilization. The band assignments of the functional groups involved in AgNPs synthesis were resolved according to [38,39]. The spectrum of AgNPs-TS (Figure 6A) was found to be comparable to the spectrum reported for *T. Harzianum* [25,30]. The presence of alcohol, phenols, carbonyl, amines (both aromatic and aliphatic) and amide functional groups is observed in both spectra AgNPs-TS (Figure 6A,B) and the report of Ahluwalia et al. [30].

In the case of FTIR analysis for AgNPs synthesized using *G. sessile*, no previous studies report the production of metallic NPs or the analysis of the metabolites produced by this fungus. Recently, *G. sessile* was reported as a fast polysaccharide producer, achieving the highest biomass and polysaccharide yields, both in liquid culture and solid-state fermentation, compared with *Ganoderma lingzhi* and *Ganoderma oregonense* [40]. Thus, this fungus has high potential for the manufacture of dietary supplements [40] and for its use in the production of metallic NPs. Previous FTIR analyses on metallic NPs synthesized with other *Ganoderma* spp. confirm the presence of capping biomolecules that stabilize and affect the morphology of the nanostructures [36,37,40,41,42,43,44,45,46,47]. Strong bands in the region of 3650–1000 cm^−1^ were reported for AgNPs synthesized using *G. lucidum* [45], with the –OH and C–OH stretching vibrations of polysaccharides represented by the appearance of a strong absorption band at 1043 cm^−1^. The metabolites responsible for the reduction of silver ions were polyphenols, polysaccharides, triterpenoids and sterols [45]. For AgNPs synthesized with *G. sessile*, the functional groups detected could be associated with proteins, aromatic amines and polysaccharides, which could be acting as reducing or/and capping agents.

As mentioned earlier, silver nanoparticles possess antimicrobial activity, and those synthesized using supernatants of *Trichoderma* spp. have been reported as possessing excellent antibacterial properties [26,30,48]. In this work, AgNPs synthesized using intracellular and extracellular components had similar antibacterial properties; in general, bacterial growth was significantly reduced in a dose-dependent manner. Usually, very low concentrations were necessary for bacterial inhibition; depending on the bacterial strain and the type of AgNPs, the necessary concentration for inhibition (MIC) was 1.26–5.0 µg/mL. Similarly, it was reported that AgNPs synthesized with the supernatant of *T. harzianum* were effective against *Staphylococcus aureus* and *Klebsiella aeruginosa.* In addition, it was found that bacterial growth was reduced in a dose-dependent manner using concentrations of 3–15 µg/mL [30]. AgNPs synthesized with intracellular components of *T. harzianum* have not been evaluated against human pathogens; however, they were evaluated against *Clavibacter michiganensis* subsp. *michiganensis*, which is the causative pathogen of tomato canker disease. The synthesized AgNPs were used for antimicrobial assays using the disk diffusion method, and the inhibitory effect improved with increased concentration of silver [26].

Studies reporting AgNPs synthesized using *Ganoderma* spp. have documented inhibitory properties against pathogenic bacteria. However, most studies for the synthesis of AgNPs using *Ganoderma* spp. have been carried out with *G. lucidum* [32,42,43,45,46,47]. Strong bactericidal activity against *S. aureus*, *E. coli*, *Staphylococcus* mutants, *Klebsilla pneumoniae* and *P. aeruginosa* was reported using AgNPs (5–30 nm) synthesized with the supernatant of *G. lucidum* [42]. Recently, other *Ganoderma* spp. have started to be investigated for their use in AgNPs synthesis and their potential applications as antimicrobial agents. For instance, AgNPs synthesized using *G. applanatum* (20–25 nm) exhibited high antioxidant capacity and in vitro antibacterial activity against *S. aureus* and *E. coli* [41]. AgNPs obtained using *G. sessiliforme* extract were effective against common food-borne bacteria, such as *E. coli, Bacillus subtilis, Streptococcus faecalis, Listeria innocua* and *Micrococcus luteus*, and it was concluded that the synthesized AgNPs could be used to control the growth of food-borne pathogens and thus have potential application in the food packaging industry [44]. Most studies with AgNPs synthesized using *Ganoderma* spp. have been carried out using the pulverized fruiting bodies to obtain the extract; however, *G. lucidum* mycelia has also been used for the synthesis of AgNPs, and strong bactericidal activity against *S. aureus* and *E. coli* was reported for AgNPs in the range of 10–70 nm [43]. Furthermore, the best inhibition of *E. coli*, *S. aureus* and *P. aeruginosa* was found with AgNPs (11.38 ± 5.51 nm) biosynthesized from *G. lucidum* extracts, compared with AgNPs synthesized by chemical methods [45]. In this work, AgNPs synthesized using *G. sessile* displayed strong bacterial inhibition at very low silver concentrations, and inhibition increased with NPs synthesized with the supernatant fraction. Furthermore, inhibition of *S. aureus* was higher with *G. sessile* AgNPs, and therefore we can assume that AgNPs are more effective to kill Gram-positive bacteria.

Although the exact mechanism of action of AgNPs on bacteria is not completely described, there is significant information on the interaction of AgNPs with bacteria and their effects. In general, the antibacterial activity of AgNPs apparently is closely related with the continuous release of Ag+; small AgNPs attach to the bacterial cell surface and release high concentrations of Ag+, causing physical modifications in the membrane [48]. AgNPs together with the continuous release of Ag+ inactivate several cell functions, causing: (a) Disruption of the cell wall and membrane, (b) Denaturation of ribosomes, inhibiting protein synthesis, (c) Interruption of ATP production, (d) Membrane disruption by ROS, (e) DNA damage, (f) Denaturation of membrane, and (g) Perforation of membrane [49]. In fact, disruption of the cell membrane, resulting in big gaps or holes in the cell surface and the consequent leakage of cellular materials, has been demonstrated [50,51]. ROS production by metallic NPs correlates with particle size, shape, surface area and chemistry [52]. High concentrations of ROS (including superoxide anion radicals, hydroxyl radicals and hydrogen peroxide) in bacterial cells can result in oxidative stress. It is reported that bacterial cells exposed to AgNPs causes overproduction of ROS attacking membrane lipids, leading to a breakdown of the membrane function [52]. ROS formation has been previously documented in bacteria when exposed to fungal synthesized AgNPs [53,54,55]. ROS production completely inhibited bacterial growth provoking membrane disruption, causing leakage of cell components and finally cell death [54,55]. In this work we also detected overproduction of ROS correlated with the bactericidal effect in bacteria exposed to different AgNPs concentrations, corroborating previous reports [53,54,55]. Cell viability assays also corroborated that very low concentrations of the biosynthesized AgNPs are necessary to inhibit bacteria and that AgNPs-GS have great potential for killing both, Gram-negative and Gram-positive bacteria.

Regarding human cell viability, we found that the pure extracts and supernatants of *T. harzianum* and *G. sessile* did not affect the viability of fibroblasts or macrophages (Figure 10). Therefore, cytotoxic effects on cell lines were due only to AgNPs, which were found to be dose-dependent. In the case of AgNPs obtained from the extract of *T. harzianum*, fibroblasts were more sensitive, presenting 80% of viability at a concentration of 2.53 µg/mL, and at higher concentrations, cell viability was up to 70 % (Figure 10A). AgNPs obtained from *T. harzianum* supernatant induced good cell viability at the higher concentrations tested (Figure 10B). Similarly, it was reported that AgNPs using the supernatant of *T. harzianum* presented cytotoxic and genotoxic effects depending on the cell line used and the exposure concentration [56]. In addition, cell lines respond differently when exposed to silver nanoparticles, some being more sensitive than others [57].

AgNPs synthesized using *G. sessile* had more cytotoxic effects in both cell lines than those synthesized using *T. harzianum*. In this case, macrophages were more susceptible than fibroblasts at the higher concentrations assayed (Figure 10B). The results obtained could be due to the smaller sizes obtained with the extracts and supernatants of *G. sessile*. In general, smaller AgNPs have been reported with more cytotoxic effects, as documented by Yen et al. [58], who reported that AgNPs of 3 nm showed more significant cytotoxicity compared to those of 25 nm at a concentration of 10.0 µg/mL in macrophage cell lines (J774 A1). AgNPs synthesized with the supernatant of *G. sessiliforme* were evaluated in fibroblast cells (L-929), and 80% of cell viability was found at high concentrations of silver (100 µg/mL); however, AgNPs had an average size of 45 nm [44].

An important characteristic of the obtained AgNPs is their stability; however, few studies document this parameter. In the case of *T. harzianum*, AgNPs were reported to be stable for 3 months [30]. In this study, all synthesized AgNPs were stable for more than 1 year, which is advantageous for increasing their shelf life.

## 5. Conclusions

A high reproducible and optimized protocol for the synthesis of small AgNPs using extracellular (supernatants) and intracellular (extracts) fungal components is reported. The results presented here suggest a harmless use of the extracts and supernatants of *T. harzianum* and *G. sessile* to synthesize nanoparticles, since no cytotoxic effects were detected in fibroblasts and macrophages. The resulting AgNPs were smaller using the supernatants of both fungi, and in general, NPs synthesized with *G. sessile* were smaller than those synthesized with *T. harzianum* (sizes were 9.6 and 19.1 nm for *T. harzianum* and 5.4 and 8.9 nm for *G. sessile* using the supernatant and extract, respectively). An important result is the low concentration of silver that was necessary for bacterial inhibition, with an MIC varying from 1.26–5.0 µg/mL and MBC from 2.5–10.0 µg/mL. Results indicate that AgNPs synthesized with *G. sessile* supernatant (AgNPs-GS) are effective against Gram-positive bacteria, killing *S. aureus* at a concentration of 10.0 µg/mL. In addition, the obtained AgNPs were stable for more than 1 year at ambient temperature, increasing their shelf life. Therefore, the potential application of AgNPs as topical antimicrobials can be further evaluated since the synthesized AgNPs preserve their antibacterial activity after a long time of storage.

## Figures and Tables

**Table 1:** Characterization of silver nanoparticles obtained with the optimized protocol, using a ratio of 1:3 *v/v* of reduction agent/AgNO_3_ 1mM and incubation time of three days at 60 °C.

Fungus	Reduction Agent	UV-Vis Peak (nm)	Average Size (nm)	Size Range (nm)	Z Potential (mV)	Hydrodynamic Diameter (nm)
*Trichoderma harzianum*	Supernatant	450	9.6	1–33	−18.5	22
	Extract	451	19.1	3–59	−11.8	33
*Ganoderma sessile*	Supernatant	435	5.4	1–25	−23.3	24
	Extract	437	8.9	1–38	−33.2	47

**Table 2:** FTIR spectrum absorption bands and the corresponding functional groups involved in the synthesis of AgNPs.

AgNPs-TS	AgNPs-TE	AgNPs-GS	AgNPs-GE	Band Assignment
3257	3244	3324	3621	N–H and O–H stretching
2916	2914	2918	2913	C–H stretching
-	-	2157	2158	C=C conjugated and C≡ stretch
-	-	2010	2023	C≡ C–H stretch
1618	1620	1608	-	C=C stretching and N–H bending
1370	1386	1348	-	–C–N stretching (Aromatic amines)
1037	1015	1037	1032	C–O bending and C–N stretching
-	-	762	766	C–H (rocking) and N–H rocking

TS = *T. harzianum* supernatant, TE = *T. harzianum* extract, GS = *G. sessile* supernatant, GE = *G. sessile* extract.

**Table 3:** Minimum inhibitory concentration (MIC) and minimum bactericidal concentration (MBC) of AgNPs synthesized with the supernatants and extracts of *T. harzianum* and *G. sessile*.

	MIC (µg/mL)				MBC (µg/mL)			
Bacterial Strains	AgNPs-TS	AgNPs-TE	AgNPs-GS	AgNPs-GE	AgNPs-TS	AgNPs-TE	AgNPs-GS	AgNPs-GE
*E. coli*	1.26	1.26	1.26	2.5	2.5	2.5	2.5	5.0
*P. aeruginosa*	1.26	1.26	1.26	1.26	2.5	2.5	2.5	2.5
*S. aureus*	5.0	5.0	2.5	5.0	10.0	10.0	10.0	10.0

## Supplementary Materials

The following supporting information can be downloaded at: https://www.mdpi.com/article/10.3390/antibiotics11060800/s1.
