# Supplementary material for: Optimized Synthesis of Small and Stable Silver Nanoparticles Using Intracellular and Extracellular Components of Fungi: An Alternative for Bacterial Inhibition
Source: Antibiotics (Basel). 2022 Jun 14;11(6):800. doi: 10.3390/antibiotics11060800 (PMC9220004; doi:10.3390/antibiotics11060800)
Supplement: Supplementary file 1 [file antibiotics-11-00800-s001.zip › antibiotics-1733680-supplementary.pdf]

antibiotics

MDPI

Supplementary Material

Results obtained and considered for optimized protocol

1. Biomass required for the synthesis of AgNPs using supernatants

In order to optimize the synthesis, different amounts of *T. harzianum* biomass (2.5, 5 and 10 g) were used to obtain supernatants. The resulting supernatants were utilized to synthesize of AgNPs using a ratio of 1:1 (reducing agent/ 5mM $AgNO_3$) and incubated at room temperature. After 24 h, the characteristic change of color from clear to light brown occurred in all reactions. By UV-Vis spectroscopy the characteristic curves of absorbance between 400-500 nm, associated with the typical Surface Plasmon Resonance (SPR) for silver nanoparticles were obtained, indicating successfully formation of AgNPs (Figure 1A). However, absorbance was higher using 10 g of biomass compared with 2.5 and 5 g, indicating a higher concentration of AgNPs. Therefore 10 g of fungal biomass, incubated in 100 mL of deionized water were used to obtain the supernatant for subsequent optimization protocol. After the incubation time, AgNPs obtained using 10 g of biomass were analyzed by TEM and NPs obtained were quasi-spherical in shape (Figure 1B) with an average size of 3.8±1.0 nm and range size of 1 to 7 nm (Figure 1C).

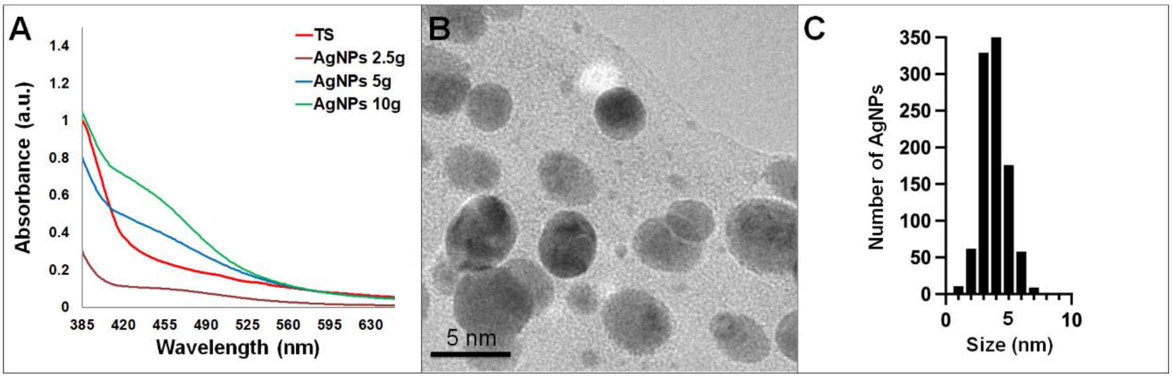

Figure S1. Synthesis of AgNPs after 24 h at room temperature using the supernatant of *T. harzianum*. (A) UV-Vis absorption curves obtained using the supernatants of different amounts of biomass and 5 mM $AgNO_3$, (B) TEM micrograph of AgNPs obtained using the supernatant of 10 g of biomass, (C) corresponding size distribution histogram. TS = *Trichoderma* supernatant.

2. Synthesis of AgNPs using fungal supernatant at different concentrations of $AgNO_3$

The synthesis of AgNPs using the supernatant of *T. harzianum* (from 10 g of biomass incubated in deionized water) was carried out at ambient temperature using different concentrations of $AgNO_3$ and different proportions of both the supernatant and the metallic precursor. The samples were first analyzed by UV-Vis spectroscopy and the absorption bands obtained are shown in Figure 2. We determined that using 1 mM of $AgNO_3$, the formation of NPs was more evident using a 1:3 ratio (supernatant, $AgNO_3$) (Figure 2A), while using a concentration of 5 mM, a similar curve of absorbance was obtained with a 1:1 ratio (Figure 2D). Analysis by TEM revealed that small quasi-spherical NPs were produced using both concentrations of $AgNO_3$ (Figure 2B, 2E); however, using 1 mM slightly larger NPs were obtained. Average size for obtained NPs using 1 mM of $AgNO_3$ and a ratio of 1:3 was 6.5 ± 5.1 nm in size, with a size range of 1 to 25 nm (Figure 2C). The synthesis of nanoparticles using a concentration of 5 mM and a ratio of 1:1, produce particles with an average size of 3.5 nm ± 2.7 nm in size, and a size range of 1 to 16 nm (Figure 2F). For subsequent syntheses, a ratio of 1:3 (reducing agent, 1 mM $AgNO_3$) was used, since similar results in size and shape were obtained using 1 and 5 mM of $AgNO_3$.

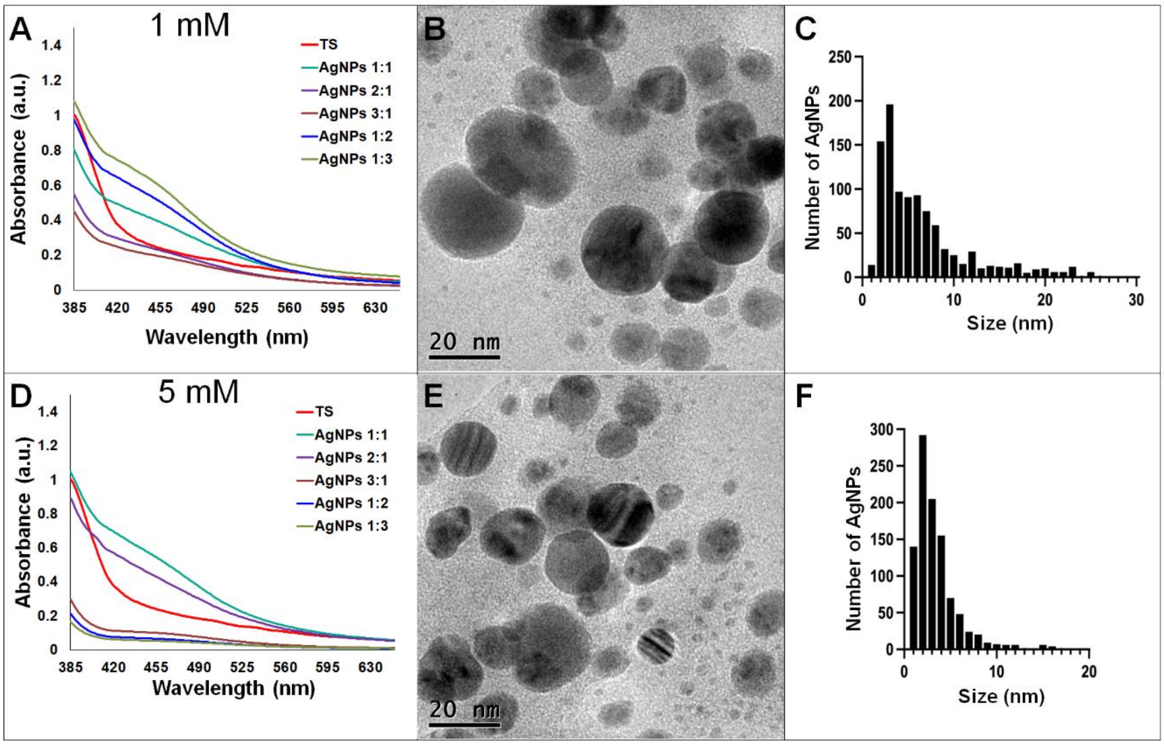

Figure S2. Synthesis of AgNPs at room temperature using the supernatant of *T. harzianum* and different proportions of supernatant:$AgNO_3$. (A-C) Results obtained using 1mM $AgNO_3$; (D-F) Results obtained using 5mM $AgNO_3$. (A, D) UV-Vis absorption curves of AgNPs; (B, E) TEM micrographs of AgNPs obtained using proportions (1:3) and (1:1), respectively and (C, F) corresponding size distribution histograms. TS = *Trichoderma* supernatant.

3. Synthesis of AgNPs using fungal supernatant at different temperatures

The synthesis of AgNPs at ambient temperature was monitored every day by UV-Vis spectroscopy, and it was after seven days that no further change was observed (data not shown). Therefore, in order to accelerate the reaction, the supernatant of *T. harzianum* was used to carry out the synthesis at different temperatures, using a ratio of 1:3 and 1 mM $AgNO_3$. Higher absorbance was detected when using 60ºC (Figure 3A). Analysis by TEM of NPs obtained at this temperature revealed quasi-spherical NPs (Figure 3B), with an average size of 7.1 ± 5.9 nm and a size range of 1–30 nm (Figure 3C). Therefore, subsequent syntheses were carried out at 60ºC.

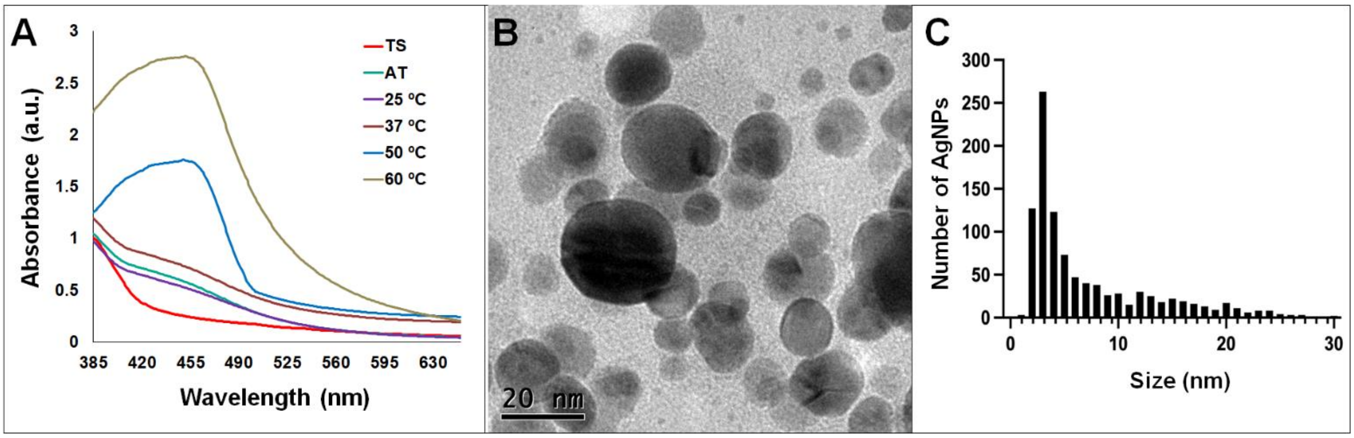

Figure S3. Synthesis of silver nanoparticles using the supernatant of *T. harzianum* at different temperatures. (A) UV-Vis absorption curves obtained at different temperatures, (B) TEM micrographs

of AgNPs synthesized at 60ºC, (C) corresponding size distribution histogram. TS = *Trichoderma* supernatant, AT = ambient temperature.
